# Supplementary material for: Establishing an empirical cut-off on the 12-item Brief Berger HIV Stigma Scale to screen psychosocial vulnerability among PLHIV in Nigeria
Source: PLOS Glob Public Health. 2026 Mar 19;6(3):e0005253. doi: 10.1371/journal.pgph.0005253 (PMC13001978; doi:10.1371/journal.pgph.0005253)
Supplement: S1 Checklist — Completed checklist indicating the page numbers or sections where each of the 22 STROBE items is reported in the manuscript. https://zenodo.org/records/17088926 (DOCX) [file pgph.0005253.s001.docx]

STROBE Checklist

**Title:** An empirical cut-off for the Brief Berger HIV Stigma Scale to identify psychosocial vulnerability in people living with HIV in Nigeria: A cross-sectional study

|  | Item No | Recommendation | Pages |
| --- | --- | --- | --- |
| **Title and abstract** | 1 | 1. Indicate the study’s design with a commonly used term in the title or the abstract   *A cross-sectional study" clearly stated in title* | 1 |
|  |  | 1. Provide in the abstract an informative and balanced summary of what was done and what was found   *Un-structured abstract covers background, methods, results, conclusions* | Page 1 |
| Introduction | | |  |
| Background/rationale | 2 | Explain the scientific background and rationale for the investigation being reported  *Introduction* | Page 2 |
| Objectives | 3 | State specific objectives, including any prespecified hypotheses  *End of Introduction* | Page 2 |
| Methods | | |  |
| Study design | 4 | Present key elements of study design early in the paper  Methods: Immediately after Study Location | Page 3 |
| Setting | 5 | Describe the setting, locations, and relevant dates, including periods of recruitment, exposure, follow-up, and data collection  *Study Location* | Page 3 |
| Participants | 6 | 1. Give the eligibility criteria, and the sources and methods of selection of participants   *Eligibility Criteria & Sampling* | Page 3 |
| Variables | 7 | Clearly define all outcomes, exposures, predictors, potential confounders, and effect modifiers. Give diagnostic criteria, if applicable  *Variables description* | Page 4 |
| Data sources/ measurement | 8* | For each variable of interest, give sources of data and details of methods of assessment (measurement). Describe comparability of assessment methods if there is more than one group  Description of Data Collection Tool | Page 4 |
| Bias | 9 | Describe any efforts to address potential sources of bias  *Included in the Methods on* | Page 6 |
| Study size | 10 | Explain how the study size was arrived at  *Included in the Methods on* | Page 4 |
| Quantitative variables | 11 | Explain how quantitative variables were handled in the analyses. If applicable, describe which groupings were chosen and why  *Included in the Methods* | Page 4 |
| Statistical methods | 12 | 1. Describe all statistical methods, including those used to control for confounding 2. *Included in the Methods & Results* | Page 5 |
|  |  | 1. Describe any methods used to examine subgroups and interactions   *Not applicable* |  |
|  |  | (*c*) Explain how missing data were addressed | Page 5 |
|  |  | 1. If applicable, describe analytical methods taking account of sampling strategy |  |
|  |  | 1. Describe any sensitivity analyses   *Not applicable* | Page 5 |
| Results | | |  |
| Participants | 13* | (a) Report numbers of individuals at each stage of study—eg numbers potentially eligible, examined for eligibility, confirmed eligible, included in the study, completing follow-up, and analysed | Page 6 |
|  |  | (b) Give reasons for non-participation at each stage | Page 6 |
|  |  | (c) Consider use of a flow diagram | Appendix |
| Descriptive data | 14* | 1. Give characteristics of study participants (eg demographic, clinical, social) and information on exposures and potential confounders   *Included in the Results* | Page 6 |
|  |  | 1. Indicate number of participants with missing data for each variable of interest   No missing data | - |
| Outcome data | 15* | Report numbers of outcome events or summary measures  *Included in the Results* | Results page 7-13 |
| Main results | 16 | 1. Give unadjusted estimates and, if applicable, confounder-adjusted estimates and their precision (eg, 95% confidence interval). Make clear which confounders were adjusted for and why they were included   *Included in the Results* | Page 12-13 |
|  |  | 1. Report category boundaries when continuous variables were categorized   *Included in the Results* | Page 7 Methods/Results |
|  |  | (*c*) If relevant, consider translating estimates of relative risk into absolute risk for a meaningful time period | Not Applicable |
| Other analyses | 17 | Report other analyses done—eg analyses of subgroups and interactions, and sensitivity analyses | Results page 12 |
| Discussion | | |  |
| Key results | 18 | Summarise key results with reference to study objectives | Page 15 |
| Limitations | 19 | Discuss limitations of the study, taking into account sources of potential bias or imprecision. Discuss both direction and magnitude of any potential bias  *Included in the Discussion on page* | Page 16 |
| Interpretation | 20 | Give a cautious overall interpretation of results considering objectives, limitations, multiplicity of analyses, results from similar studies, and other relevant evidence  *Included in the Discussion on page* | Page 15-16 |
| Generalisability | 21 | Discuss the generalisability (external validity) of the study results  *Included in the Discussion on page* | Page 15-16 |
| Other information | | |  |
| Funding | 22 | Give the source of funding and the role of the funders for the present study and, if applicable, for the original study on which the present article is based | End of manuscript  Page 17 |
