# Supplementary material for: Establishing an empirical cut-off on the 12-item Brief Berger HIV Stigma Scale to screen psychosocial vulnerability among PLHIV in Nigeria
Source: PLOS Glob Public Health. 2026 Mar 19;6(3):e0005253. doi: 10.1371/journal.pgph.0005253 (PMC13001978; doi:10.1371/journal.pgph.0005253)
Supplement: S1 Data — Anonymized participant-level data used for analyses, including stigma scale responses, psychosocial vulnerability indicators, sociodemographic variables, and clinical measures (n = 285). [31] Available from https://zenodo.org/records/17088926 (DOCX) [file pgph.0005253.s010.docx]

**Supplementary Table 8. Test for interaction between total stigma score and disclosure status in predicting psychosocial vulnerability**

| **Term** | **Coefficient** | **Std. Error** | **z-value** | **p-value** |
| --- | --- | --- | --- | --- |
| (Intercept) | -4.558 | 1.613 | -2.826 | 0.0047 |
| Stigma Score | 0.124 | 0.045 | 2.724 | 0.0065 |
| Disclosure status (ref: Disclosed) |  |  |  |  |
| Non-disclosed | 0.076 | 1.818 | 0.042 | 0.967 |
| Stigma Score × Non-disclosed (interaction) | 0.004 | 0.052 | 0.070 | **0.945** |
