# Supplementary material for: Establishing an empirical cut-off on the 12-item Brief Berger HIV Stigma Scale to screen psychosocial vulnerability among PLHIV in Nigeria
Source: PLOS Glob Public Health. 2026 Mar 19;6(3):e0005253. doi: 10.1371/journal.pgph.0005253 (PMC13001978; doi:10.1371/journal.pgph.0005253)
Supplement: S1 Table — Presents item means and standard deviations, average interitem covariance, subscale mean scores, and Cronbach’s alpha reliability coefficients for the four stigma dimensions (Personalized Stigma, Disclosure Concerns, Concerns about Public Attitudes, and Negative Self-Image). (DOCX) [file pgph.0005253.s002.docx]

An empirical cut-off for the Brief Berger HIV Stigma Scale to identify psychosocial vulnerability in people living with HIV in Nigeria: A cross-sectional study

Fig 1: Flowchart
