# Supplementary material for: Establishing an empirical cut-off on the 12-item Brief Berger HIV Stigma Scale to screen psychosocial vulnerability among PLHIV in Nigeria
Source: PLOS Glob Public Health. 2026 Mar 19;6(3):e0005253. doi: 10.1371/journal.pgph.0005253 (PMC13001978; doi:10.1371/journal.pgph.0005253)
Supplement: S2 Table — Shows the frequency and percentage of participants classified as having high versus low stigma across the four stigma subscales. (DOCX) [file pgph.0005253.s003.docx]

The 12-item Berger HIV Stigma scale measures four subscales: Personalized Stigma, Disclosure Concerns, Concern about Public Attitude and Negative Self-Image. The overall scale reliability is strong (0.87), and participants reported varying levels of stigma across the subscales. While the average interitem covariance is moderate (0.27), the scale demonstrates good internal consistency among participants.

**Supplementary Table 1. Descriptive statistics for items and subscales in the short-form version of the Berger Human Immunodeficiency Virus stigma scale**

|  | **Mean item score (SD)** | **Average interitem covariance** | **Mean subscale score (SD)** | **Reliability α** |
| --- | --- | --- | --- | --- |
| 12 - item Berger HIV Stigma scale |  | 0.27 | 33.62 (6.74) | 0.87 |
| **Personalized Stigma** |  | 0.44 | 5.38 (1.47) | 0.77 |
| Some people avoid touching me once they know I have HIV | 2.86 (0.79) |  |  |  |
| People I care about stopped calling after learning I have HIV | 2.21 (0.96) |  |  |  |
| I have lost friends by telling them I have HIV | 2.19 (0.95) |  |  |  |
| **Disclosure Concerns** |  | 0.25 | 9.91 (1.35) | 0.72 |
| Telling someone I have HIV is risky | 2.97 (0.87) |  |  |  |
| I work hard to keep my HIV a secret | 3.16 (0.69) |  |  |  |
| I am very careful who I tell that I have HIV | 3.24 (0.63) |  |  |  |
| **Concerns about public attitudes** |  | 0.27 | 7.66 (1.37) | 0.62 |
| People with HIV are treated like outcasts | 3.05 (0.79) |  |  |  |
| Most people believe that a person who has HIV is dirty | 2.63 (1.07) |  |  |  |
| Most people are uncomfortable around someone with HIV | 3.24 (0.72) |  |  |  |
| **Negative self-image** |  | 0.41 | 4.74 (0.85) | 0.72 |
| I feel guilty because I have HIV | 2.65 (0.80) |  |  |  |
| People's attitudes about HIV make me feel worse about myself | 2.89 (0.80) |  |  |  |
| I feel I am not as good a person as others because I have HIV | 2.47 (1.14) |  |  |  |
