# Supplementary material for: Establishing an empirical cut-off on the 12-item Brief Berger HIV Stigma Scale to screen psychosocial vulnerability among PLHIV in Nigeria
Source: PLOS Glob Public Health. 2026 Mar 19;6(3):e0005253. doi: 10.1371/journal.pgph.0005253 (PMC13001978; doi:10.1371/journal.pgph.0005253)
Supplement: S3 Table — Displays the number of true positives, true negatives, false positives, and false negatives when using a cut-off score of ≥30 to identify psychosocial vulnerability (n = 285). (DOCX) [file pgph.0005253.s004.docx]

The perceived HIV stigma subscale data show that a majority of participants reported high stigma for Disclosure Concern (96.14%) and Public Attitude (56.49%). In contrast, Personalized Stigma was high for 6.67% of participants, while Negative Self-Image was high for only 0.35% (n=1), indicating that concerns about disclosure and public attitudes are more prevalent than internalized or personalized stigma

**Supplementary Table 2: Perceived Human Immunodeficiency Virus stigma subscale.**

| **Stigma Subscale** | **High**  **Freq (%)** | **Low**  **Freq (%)** |
| --- | --- | --- |
| Personalized | 19 (6.67) | 266 (93.33) |
| Disclosure concern | 273 (96.14) | 11 (3.86) |
| Public attitude | 161 (56.49) | 124 (43.51) |
| Negative self-image | 1 (0.35) | 284 (99.65) |
