# Supplementary material for: Establishing an empirical cut-off on the 12-item Brief Berger HIV Stigma Scale to screen psychosocial vulnerability among PLHIV in Nigeria
Source: PLOS Glob Public Health. 2026 Mar 19;6(3):e0005253. doi: 10.1371/journal.pgph.0005253 (PMC13001978; doi:10.1371/journal.pgph.0005253)
Supplement: S4 Table — Reports positive and negative predictive values for hypothetical prevalence rates of 10% and 25%, based on the study’s sensitivity (87.5%) and specificity (49.0%). (DOCX) [file pgph.0005253.s005.docx]

Two-by-two classification table showing the performance of the 12-item Brief Berger HIV Stigma Scale using a cut-off score of ≥30 for identifying psychosocial vulnerability (n=285). At this threshold, the scale correctly identified 112 of 128 vulnerable participants (true positives) and 77 of 157 non-vulnerable participants (true negatives), yielding a sensitivity of 87.5% and specificity of 49.0%.

**Supplementry Table 3: Confusion matrix for the 12-item Brief Berger HIV Stigma Scale at cut-off ≥30**

|  | **Psychosocial Vulnerability Present (n=128)** | **Psychosocial Vulnerability Absent (n=157)** | **Total** |
| --- | --- | --- | --- |
| Stigma score ≥30 | 112 (True Positive) | 80 (False Positive) | 192 |
| Stigma score <30 | 16 (False Negative) | 77 (True Negative) | 93 |
| Total | 128 | 157 | 285 |

S
