# Supplementary material for: Establishing an empirical cut-off on the 12-item Brief Berger HIV Stigma Scale to screen psychosocial vulnerability among PLHIV in Nigeria
Source: PLOS Glob Public Health. 2026 Mar 19;6(3):e0005253. doi: 10.1371/journal.pgph.0005253 (PMC13001978; doi:10.1371/journal.pgph.0005253)
Supplement: S5 Table — Provides bivariate comparisons of sociodemographic and clinical variables between participants with and without psychosocial vulnerability, including p-values for each comparison. (DOCX) [file pgph.0005253.s006.docx]

At lower baseline prevalence levels (10% and 25%), the ≥30 cut-off maintains a high negative predictive value while the positive predictive value declines

**Supplementary Table 4: Predictive values of the ≥30 cut-off at lower hypothetical prevalence levels**

| **Prevalence of Psychosocial Vulnerability** | **Positive Predictive Value (PPV)** | **Negative Predictive Value (NPV)** |
| --- | --- | --- |
| **10%** | 0.16 (16.0%) | 0.97 (97.2%) |
| **25%** | 0.36 (36.4%) | 0.92 (92.2%) |

*Sensitivity = 87.5%; Specificity = 49%.*
