# Supplementary material for: Establishing an empirical cut-off on the 12-item Brief Berger HIV Stigma Scale to screen psychosocial vulnerability among PLHIV in Nigeria
Source: PLOS Glob Public Health. 2026 Mar 19;6(3):e0005253. doi: 10.1371/journal.pgph.0005253 (PMC13001978; doi:10.1371/journal.pgph.0005253)
Supplement: S6 Table — Presents odds ratios, 95% confidence intervals, and p-values for associations between each of the four stigma subscales and psychosocial vulnerability. (DOCX) [file pgph.0005253.s007.docx]

Higher total of 12-item Brief Berger HIV Stigma Scale, female sex, and unemployment were significantly associated with psychosocial vulnerability in bivariate analysis, whereas age, marital status, education, household size, income, disclosure status, and duration of HIV diagnosis were not (all p ≥ 0.2 except where otherwise noted).

**Supplementry Table 5: Participant Characteristics by Psychosocial Vulnerability Status**

| **Variable** | **No Vulnerability (n=157)** | **Psychosocial Vulnerability (n=128)** | **p-value** |
| --- | --- | --- | --- |
| **Total Berger HIV Stigma Score** | 30 (27–36) | 37 (33–41) | <0.001 (Wilcoxon) |
| **Age group (years)** |  |  | 0.60 |
| 21–30 | 11 (7.0%) | 14 (10.9%) |  |
| 31–40 | 20 (12.7%) | 19 (14.8%) |  |
| 41–50 | 71 (45.2%) | 53 (41.4%) |  |
| ≥51 | 55 (35.0%) | 42 (32.8%) |  |
| **Sex** |  |  | 0.007 |
| Female | 104 (66.2%) | 103 (80.5%) |  |
| Male | 53 (33.8%) | 25 (19.5%) |  |
| **Marital status** |  |  | 0.20 |
| Married/Co-habiting | 102 (65.0%) | 90 (70.3%) |  |
| Single/Widowed | 23 (14.6%) | 22 (17.2%) |  |
| Divorced/Separated | 32 (20.4%) | 16 (12.5%) |  |
| **Level of education** |  |  | 0.20 |
| No formal education | 13 (8.3%) | 10 (7.8%) |  |
| Primary | 14 (8.9%) | 18 (14.1%) |  |
| Secondary | 60 (38.2%) | 57 (44.5%) |  |
| Tertiary | 70 (44.6%) | 43 (33.6%) |  |
| **Number of people in household** |  |  | 0.30 |
| 1–2 | 34 (21.7%) | 36 (28.1%) |  |
| 3–4 | 63 (40.1%) | 41 (32.0%) |  |
| ≥5 | 60 (38.2%) | 51 (39.8%) |  |
| **Employment status** |  |  | 0.029* |
| Full-time | 37 (23.6%) | 30 (23.4%) |  |
| Part-time | 93 (59.2%) | 63 (49.2%) |  |
| Retired | 9 (5.7%) | 4 (3.1%) |  |
| Unemployed | 18 (11.5%) | 31 (24.2%) |  |
| **Monthly income (NGN)** |  |  | 0.20 |
| <50,000 | 72 (45.9%) | 73 (57.0%) |  |
| 50,000–100,000 | 64 (40.8%) | 42 (32.8%) |  |
| >100,000 | 21 (13.4%) | 13 (10.2%) |  |
| **HIV serostatus disclosure** |  |  | 0.60 |
| Yes (to ≥1 person) | 124 (79.0%) | 104 (81.2%) |  |
| No | 33 (21.0%) | 24 (18.8%) |  |
| **Duration living with HIV (years)** |  |  | 0.30 |
| <1 | 4 (2.5%) | 6 (4.7%) |  |
| 1–4 | 15 (9.6%) | 7 (5.5%) |  |
| ≥5 | 138 (87.9%) | 115 (89.8%) |  |

*Fisher’s exact test (employed because of small expected cell counts in retired category)
