# Supplementary material for: Establishing an empirical cut-off on the 12-item Brief Berger HIV Stigma Scale to screen psychosocial vulnerability among PLHIV in Nigeria
Source: PLOS Glob Public Health. 2026 Mar 19;6(3):e0005253. doi: 10.1371/journal.pgph.0005253 (PMC13001978; doi:10.1371/journal.pgph.0005253)
Supplement: S7 Table — Reports prevalence of psychosocial vulnerability, PHQ-9 ≥ 10, GAD-7 ≥ 10, total stigma score (median, IQR), and Disclosure Concerns subscale score (median, IQR) stratified by disclosure status (disclosed to at least one non-healthcare person vs non-disclosed), with corresponding p-values. (DOCX) [file pgph.0005253.s008.docx]

All four stigma dimensions were statistically significantly associated with psychosocial vulnerability in unadjusted analysis.

**Supplementry Table 6: Univariable Logistic Regression Analyses of Associations Between Berger HIV Stigma Subscales and Psychosocial Vulnerability**

| **Stigma Subscale** | **Odds Ratio (OR)** | **95% CI** | **p-value** |
| --- | --- | --- | --- |
| Negative Self-Image | 1.81 | 1.35 – 2.45 | <0.001 |
| Concerns with Public Attitudes | 1.75 | 1.43 – 2.17 | <0.001 |
| Disclosure Concerns | 1.39 | 1.16 – 1.67 | <0.001 |
| Personalised Stigma | 1.22 | 1.03 – 1.44 | 0.019 |
