# Supplementary material for: Establishing an empirical cut-off on the 12-item Brief Berger HIV Stigma Scale to screen psychosocial vulnerability among PLHIV in Nigeria
Source: PLOS Glob Public Health. 2026 Mar 19;6(3):e0005253. doi: 10.1371/journal.pgph.0005253 (PMC13001978; doi:10.1371/journal.pgph.0005253)
Supplement: S8 Table — Presents coefficients, standard errors, z-values, and p-values from logistic regression including the interaction term (total stigma score × disclosure status), confirming no evidence of effect modification (interaction p = 0.945). (DOCX) [file pgph.0005253.s009.docx]

**Supplementary Table 7.** Comparison of psychosocial vulnerability and stigma measures by disclosure status

|  | **Disclosure Status** | |  |
| --- | --- | --- | --- |
| **Variable** | **Disclosed**, N = 57^1^ | **Non-disclosed**, N = 228^1^ | **p-value**^2^ |
| **Psychosocial vulnerability** | 42.1% (24) | 45.6% (104) | 0.7 |
| **PHQ-9 ≥10 (depression)** | 38.6% (22) | 37.3% (85) | >0.9 |
| **GAD-7 ≥10 (anxiety)** | 31.6% (18) | 35.5% (81) | 0.7 |
| **Total stigma score, median (IQR)** | 35.0 (28.0, 39.0) | 34.0 (28.0, 38.0) | 0.7 |
| **Disclosure Concerns subscale, median (IQR)** |  |  | 0.6 |
| 5 | 0.0% (0) | 0.4% (1) |  |
| 6 | 0.0% (0) | 0.4% (1) |  |
| 7 | 0.0% (0) | 3.9% (9) |  |
| 8 | 7.0% (4) | 3.1% (7) |  |
| 9 | 38.6% (22) | 37.3% (85) |  |
| 10 | 22.8% (13) | 25.4% (58) |  |
| 11 | 8.8% (5) | 11.0% (25) |  |
| 12 | 22.8% (13) | 18.4% (42) |  |
| ^1^% (n); Median (IQR) | | | |
| ^2^Pearson's Chi-squared test; Wilcoxon rank sum test | | | |
